# Darolutamide-based triple therapy combined with PSMA imaging guided focal treatment in patients with prostate cancer: a multicenter retrospective study

**DOI:** 10.3389/fphar.2025.1692407

**Published:** 2025-12-03

**Authors:** Dewen Zhong, Ruochen Zhang, Jinling Wu, Zhixun Guo, Yu Zhang, Qinfei Zhou, Zijun Zou, Deng Lin, Zhonglei Lu, Jiawen Wang, Jinfeng Wu, Liefu Ye, Yongbao Wei

**Affiliations:** 1 Department of Urology, Longyan First Affiliated Hospital of Fujian Medical University, Longyan, Fujian, China; 2 Shengli Clinical Medical College of Fujian Medical University, Department of Urology, Fujian Provincial Hospital, Fuzhou University Affiliated Provincial Hospital, Fuzhou, China; 3 School of Clinical Medicine, Fujian Medical University, Fuzhou, China; 4 Department of Nuclear Medicine, Shengli Clinical Medical College of Fujian Medical University, Fujian Provincial Hospital, Fuzhou University Affiliated Provincial Hospital, Fuzhou, China; 5 Department of Pain and Rehabilitation Medicine, Zhejiang Cancer Hospital, Hangzhou Institute of Medicine (HIM), Chinese Academy of Sciences, Hangzhou, Zhejiang, China; 6 Department of Urology, Guizhou Provincial People’s Hospital, Guiyang, China; 7 College of Biological Science and Engineering, Fuzhou University, Fuzhou, Fujian, China

**Keywords:** darolutamide, triple therapy, PSMA imaging, focal treatment, metastatic hormone-sensitive prostate cancer

## Abstract

**Background:**

Darolutamide, a novel androgen receptor inhibitor, significantly improved outcomes in the ARASENS trial when combined with androgen deprivation therapy (ADT) and docetaxel chemotherapy for metastatic hormone-sensitive prostate cancer (mHSPC). In China, the regimen has been reimbursed since December 2023, expanding real-world accessibility. PSMA imaging–guided focal therapy enables precise localization of residual or oligometastatic lesions and may complement systemic control.

**Objective:**

To evaluate the real-world efficacy and safety of darolutamide-based triplet therapy combined with PSMA imaging–guided focal treatment in patients with mHSPC.

**Methods:**

This multicenter, retrospective study included 17 patients treated across three tertiary hospitals between June 2023 and June 2024. All patients received darolutamide (600 mg twice daily), ADT, and docetaxel (75 mg/m^2^ every 3 weeks for 4–6 cycles), followed 4–6 weeks later by focal therapy (surgery or radiotherapy) based on multidisciplinary team (MDT) assessment and PSMA IMAGING findings. Data collection followed STROBE recommendations. The primary endpoint was PSA90 response; secondary endpoints included PSA ≤0.2 ng/mL, PSA progression-free survival (PSA-PFS), radiographic PFS (rPFS), and safety.

**Results:**

Seventeen patients with predominantly low- to intermediate-volume metastatic disease were analyzed. The PSA90 response rate was 94.1%, and 82.4% achieved PSA ≤0.2 ng/mL. Median PSA-PFS and rPFS were not reached at a median follow-up of 16 months. The 12-/18-month PSA-PFS rates were 88% and 65%, while the corresponding rPFS rates were 94% and 76%. Grade 1–2 myelosuppression (64.7%) and fatigue (47.1%) were most common; grade 3 neutropenia occurred in two patients (11.8%) without grade ≥4 events. Focal therapy was well tolerated, with no severe complications.

**Conclusion:**

Darolutamide-based triplet therapy combined with PSMA-guided focal intervention achieved deep biochemical responses and durable disease control with manageable toxicity in real-world Chinese patients with mHSPC. The multicenter design and standardized methodology support the feasibility of this multimodal approach, which warrants validation in larger prospective studies.

## Introduction

Prostate cancer is one of the most common urological malignancies among men in China. With the aging population and lifestyle changes, its incidence has been increasing annually, and the proportion of patients presenting with advanced disease remains high (Xia et al., 2022). In metastatic hormone-sensitive prostate cancer (mHSPC), androgen deprivation therapy (ADT) alone can achieve short-term disease control; however, most patients progress to metastatic castration-resistant prostate cancer (mCRPC) within 2–3 years, significantly compromising survival (Kyriakopoulos et al., 2018). Achieving deeper and more durable disease control in the early stages remains a major clinical challenge.

Darolutamide is a novel nonsteroidal androgen receptor inhibitor characterized by high selectivity and low blood–brain barrier penetration, thereby reducing the risk of central nervous system–related adverse events (Fizazi et al., 2019). The phase III ARASENS trial demonstrated that adding darolutamide to ADT and docetaxel significantly prolonged overall survival in patients with mHSPC, while also improving multiple secondary endpoints (Smith et al., 2022). This trial established the evidence base for triple therapy (darolutamide + ADT + docetaxel). In December 2023, China included the darolutamide–docetaxel combination regimen in the national health insurance formulary, greatly enhancing its clinical accessibility and facilitating its widespread use, thereby creating opportunities to evaluate its real-world efficacy and safety.

In recent years, the concept of multimodal therapy for mHSPC has gained increasing attention. Several studies suggest that, after systemic therapy achieves control of disseminated disease, targeted local interventions such as focal surgery (prostatectomy, etc.) or radiotherapy for residual or dominant lesions can further delay progression and improve survival outcomes (Boeve et al., 2019; Parker et al., 2018; Heidenreich et al., 2015). However, optimal patient selection and accurate target identification for focal treatment remain critical clinical challenges. Prostate-specific membrane antigen (PSMA) imaging such as positron emission tomography/computed tomography (PSMA PET/CT), currently the most sensitive molecular imaging modality for prostate cancer, enables high-resolution visualization of disease distribution throughout the body. It is particularly advantageous in detecting small-volume metastases that are often missed by conventional imaging, thereby providing a robust basis for individualized focal treatment planning (Hofman et al., 2020; Zhang et al., 2022; Fendler et al., 2016).

Although darolutamide-based triple therapy and PSMA IMAGING–guided focal interventions each have supporting evidence from randomized controlled trials, there is a paucity of systematic data regarding their combined application in the real-world setting. Therefore, we conducted a multicenter retrospective analysis to evaluate the disease control efficacy, safety, and feasibility of this combination approach in mHSPC patients within the context of expanded reimbursement coverage. Our findings aim to provide clinical evidence for optimizing multimodal treatment strategies.

## Methods

### Study design and ethical approval

This was a multicenter, retrospective, observational clinical study conducted jointly by Fujian Provincial Hospital, Longyan First Hospital, and Zhejiang Cancer Hospital—three tertiary academic medical centers in China. The study period was from June 2023 to June 2024, with follow-up until August 2025. The study protocol was approved by the institutional ethics committees of our center. Given the retrospective nature of the analysis, the requirement for informed consent was waived (von Elm et al., 2007). Although treatment decisions were made independently at each institution, all participating centers followed national and international guideline–based standards for the management of mHSPC, ensuring comparable treatment frameworks across sites. Chemotherapy cycles (docetaxel 75 mg/m^2^ every 3 weeks for 4–6 cycles), darolutamide dosing (600 mg twice daily), and focal therapy indications were highly consistent among centers. PSMA imaging modalities (^68Ga-PSMA PET/CT or ^99mTc-PSMA SPECT/CT) were applied based on local equipment availability, and all scans were retrospectively reviewed by two senior nuclear medicine physicians to maintain inter-center consistency. These retrospective harmonization measures minimized institutional variability and improved the comparability of pooled analyses. To further minimize potential biases, uniform inclusion and exclusion criteria were applied across all participating centers, standardized definitions of treatment exposure, efficacy endpoints, and adverse events were used, and data collection followed the STROBE recommendations. A detailed patient flow diagram was presented to ensure transparent case selection and attrition tracking.

The inclusion criteria were as follows: (a) Pathologically confirmed metastatic prostate adenocarcinoma (clinical stage T1–T4, N0–1, M1), presenting as bone metastases, lymph node metastases, or oligometastatic disease (≤5 lesions). (b) All patients received first-line systemic treatment with darolutamide (600 mg twice daily) in combination with androgen deprivation therapy (ADT) and docetaxel chemotherapy (75 mg/m^2^ every 3 weeks), with a minimum of four cycles and up to six cycles permitted (Smith et al., 2022). (c) During chemotherapy, short-term use of a first-generation antiandrogen to prevent flare was allowed. (d) Four to 6 weeks after completing chemotherapy, all patients underwent whole-body ^68Ga-PSMA PET/CT (Fendler et al., 2016) or ^99m^Tc-PSMA SPECT/CT (Zhang et al., 2022) assessment; if residual metabolically active primary or oligometastatic lesions were detected, focal therapy was performed based on multidisciplinary team (MDT) review, including focal surgery or radiotherapy. (e) Patients had not previously received darolutamide or chemotherapy, with prior ADT duration ≤6 months. (f) Complete clinical data were available, and treatment efficacy and safety data could be obtained during the study period (enrollment from June 2023 to June 2024, follow-up until August 2025).

Exclusion criteria included: prior treatment with other novel androgen receptor inhibitors (e.g., abiraterone, enzalutamide, apalutamide) or chemotherapy exceeding 6 months; fewer than four cycles of chemotherapy or regimens inconsistent with the darolutamide + ADT + docetaxel combination; absence of PSMA IMAGING evaluation or focal therapy; concurrent active malignancies; severe cardiac, hepatic, or renal dysfunction precluding treatment; and incomplete key clinical data preventing efficacy or safety analysis.

## Treatment protocol

### Phase 1 – Systemic therapy

Following diagnosis, all patients initiated ADT with either a luteinizing hormone-releasing hormone (LHRH) agonist or antagonist, with optional short-term first-generation antiandrogen to prevent flare. Darolutamide (600 mg twice daily) was administered continuously until disease progression or unacceptable toxicity. Docetaxel (75 mg/m^2^ IV every 3 weeks) was given concurrently for a minimum of four cycles, up to six cycles based on tolerance, hematologic parameters, and MDT recommendations (Smith et al., 2022; Tannock et al., 2004). Hematologic, liver, and renal function tests, as well as adverse events, were monitored each cycle, with dose modifications or delays as needed.

### Phase 2 – Precision assessment

Four to 6 weeks post-chemotherapy, all patients underwent PSMA imaging scanning to evaluate residual disease distribution and metabolic activity. Special attention was given to the primary tumor, regional lymph nodes, and bone lesions. Lesions with significantly increased SUVmax compared to background were considered metabolically active and eligible for focal intervention (Hofman et al., 2020; Zhang et al., 2022; Fendler et al., 2016).

### Phase 3 – Focal therasectionby

Patients with active lesions identified on PSMA IMAGING underwent individualized focal interventions after MDT discussion. Those in good general condition and meeting surgical criteria underwent focal surgery such as prostatectomy (with or without pelvic lymph node dissection as indicated (Boeve et al., 2019; Parker et al., 2018)) or transurethral resection of the prostate (TURP). Patients unsuitable for surgery or preferring radiotherapy received intensity-modulated radiotherapy (IMRT) or stereotactic body radiotherapy (SBRT) targeting the primary and/or oligometastatic lesions, with dose and fractionation determined according to international guidelines and institutional protocols (Heidenreich et al., 2015). The choice of local modality was determined through MDT discussion integrating PSMA IMAGING findings, patient performance status, and anatomical feasibility. Patients with ≤5 metabolically active lesions and a dominant intraprostatic focus with SUVmax ≥8 were deemed suitable candidates for focal intervention. Those with ECOG 0–1 and technically resectable intraprostatic lesions were preferentially treated with focal surgery. In contrast, patients unfit for surgery, exhibiting multifocal pelvic involvement, or declining surgical intervention were assigned to IMRT or SBRT. Final MDT decisions considered PSMA uptake intensity, lesion size, proximity to critical structures, and patient preference to ensure individualized, evidence-based management.

### Phase 4 – Maintenance and follow-up

After focal therapy, patients continued darolutamide and ADT maintenance. Upon disease progression, treatment strategies were adjusted based on MDT evaluation. Follow-up visits occurred every 3 months, including PSA and testosterone testing, and imaging when clinically indicated. Follow-up was calculated from the initiation of systemic therapy until August 2025.

### Data collection and analysis

Baseline demographics (age, BMI, comorbidities), tumor characteristics (Gleason score, clinical stage, metastatic sites), treatment details (darolutamide duration, chemotherapy cycles, type/timing of focal therapy), efficacy endpoints (baseline and last PSA, PSA50, PSA90, PSA ≤0.2 ng/mL), and adverse events (graded per CTCAE v5.0) were collected.

The primary endpoint was PSA90 response rate. Secondary endpoints included PSA50 response rate, PSA ≤0.2 proportion, and imaging-based progression-free survival (rPFS) and PSA progression-free survival (PSA-PFS). PSA progression was defined according to the Prostate Cancer Working Group 3 (PCWG3) criteria as a ≥25% and ≥2 ng/mL increase from the PSA nadir, confirmed by a second consecutive measurement obtained at least 3 weeks later. The PSA90 response rate was chosen as the primary endpoint because a decline of ≥90% in PSA within 6 months has been validated as a surrogate marker of treatment efficacy and long-term survival in mHSPC, as demonstrated in the ARASENS and CHAARTED trials (Kyriakopoulos et al., 2018; Smith et al., 2022). This endpoint allows for rapid and objective assessment of treatment benefit in real-world clinical practice. Given that most patients had not progressed by the data cutoff, PFS was descriptively reported as “not reached.”

Statistical analyses were performed using SPSS version 26.0. Continuous variables were expressed as median (range), and categorical variables as counts (percentage). Kaplan-Meier analysis was used to estimate PSA-PFS and rPFS at 12 and 18 months (Dinse and Lagakos, 1982), and 95% confidence intervals (CIs) were calculated to describe the variability of these survival estimates.

Waterfall plots were constructed in GraphPad Prism 10.0 to visualize the maximum PSA reduction from baseline for each patient, and a flow diagram was generated to summarize the overall screening, exclusion, and treatment allocation process. These graphical analyses were designed to improve reproducibility and visual transparency of patient outcomes.

## Results

### Patient selection and baseline characteristics

**FIGURE 1:**
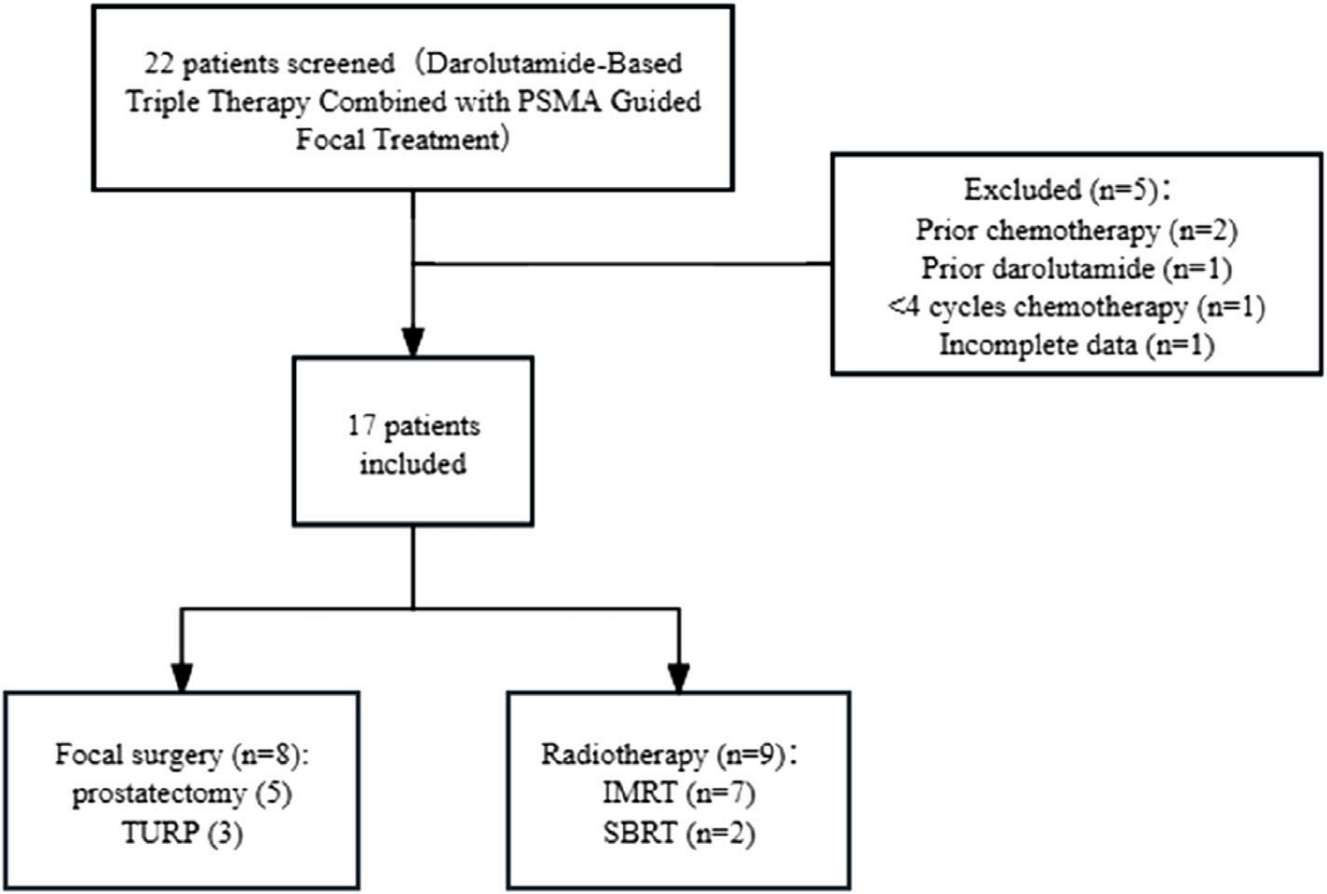
Study design and patient selection flowchart.

A detailed flow diagram summarizing patient screening, exclusion, and focal treatment allocation is presented in Figure 1. Between June 2023 and June 2024, a total of 22 eligible mHSPC patients were screened across the three participating centers (Fujian Provincial Hospital, Longyan First Hospital, and Zhejiang Cancer Hospital). Excluded cases were: prior chemotherapy (n = 2), prior darolutamide treatment (n = 1), fewer than four cycles of chemotherapy (n = 1), and incomplete key clinical data (n = 1). Seventeen patients met the inclusion criteria and were included in the final analysis.

The median age was 64 years (range, 52–78 years) and the median BMI was 23.0 kg/m^2^ (range, 20.1–26.8) (Table 1). Fourteen patients (82.4%) had a Gleason score ≥8, including 10 with a score of 8, two with 9, and two with 10. Thirteen patients (76.5%) had clinical stage T3 or higher, and seven (41.2%) were N1. Sites of metastases included bone in 11 patients (64.7%), pelvic lymph nodes in 8 (47.1%), and visceral metastases in 2 (11.8%). This distribution suggests that most enrolled patients presented with low-to intermediate-volume metastatic disease, consistent with the real-world subgroup typically considered for focal interventions in mHSPC. The most common comorbidities were hypertension (n = 5, 29.4%), diabetes mellitus (n = 3, 17.6%), and hyperlipidemia (n = 4, 23.5%).

**TABLE 1:** Baseline characteristics.

Variable	Median/n (%)
BMI (kg/m^2^)	23.0 (20.1–26.8)
Initial PSA (ng/mL)	39.7 (23.0–163.0)
Gleason score	7: 1 (5.9%); 8: 10 (58.8%); 9: 2 (11.8%); 10: 2 (11.8%)
T Stage	T2: 3; T2b: 2; T3: 1; T3b: 6; T4: 4; cTx: 1
N stage	N0: 5 (29.4%); N1: 12 (70.6%)
M Stage	M1a: 2 (11.8%); M1b: 12 (70.6%); M1c: 3 (17.6%)
Prior treatment	None: 8 (47.1%); 1 ARI: 9 (52.9%)
Duration of prior treatment (months)	3 (2–5)
Comorbidities	Hypertension: 5 (29.4%); diabetes: 2 (11.6%); hyperlipidemia: 1 (5.9%); COPD: 1 (5.9%); liver disease: 2 (11.8%)
Presence of special pathological subtypes	Yes: 3 (17.6%)
HRR mutation on genetic testing	Positive: 1 (5.9%); negative: 16 (94.1%)

Abbreviations: BMI, body mass index; PSA, prostate-specific antigen; ADT, androgen deprivation therapy; COPD, chronic obstructive pulmonary disease; HRR, homologous recombination repair.

### Systemic treatment

All patients received initial triplet therapy consisting of darolutamide (600 mg orally twice daily) combined with ADT and docetaxel chemotherapy. The median duration of darolutamide treatment was 14 months (range, 9–18 months). The median number of chemotherapy cycles was five (range, 4–6), with eight patients (47.1%) completing four cycles, one patient (5.9%) completing five cycles, and eight patients (47.1%) completing six cycles.

### PSMA imaging assessment and focal therapy

Four to 6 weeks after completing chemotherapy, all patients underwent PSMA IMAGING. Sixteen patients (94.1%) had metabolically active lesions, primarily in the prostate primary tumor (100%), seminal vesicles (35.3%), pelvic lymph nodes (70.6%), and solitary bone metastases (11.8%).

**FIGURE 2:**
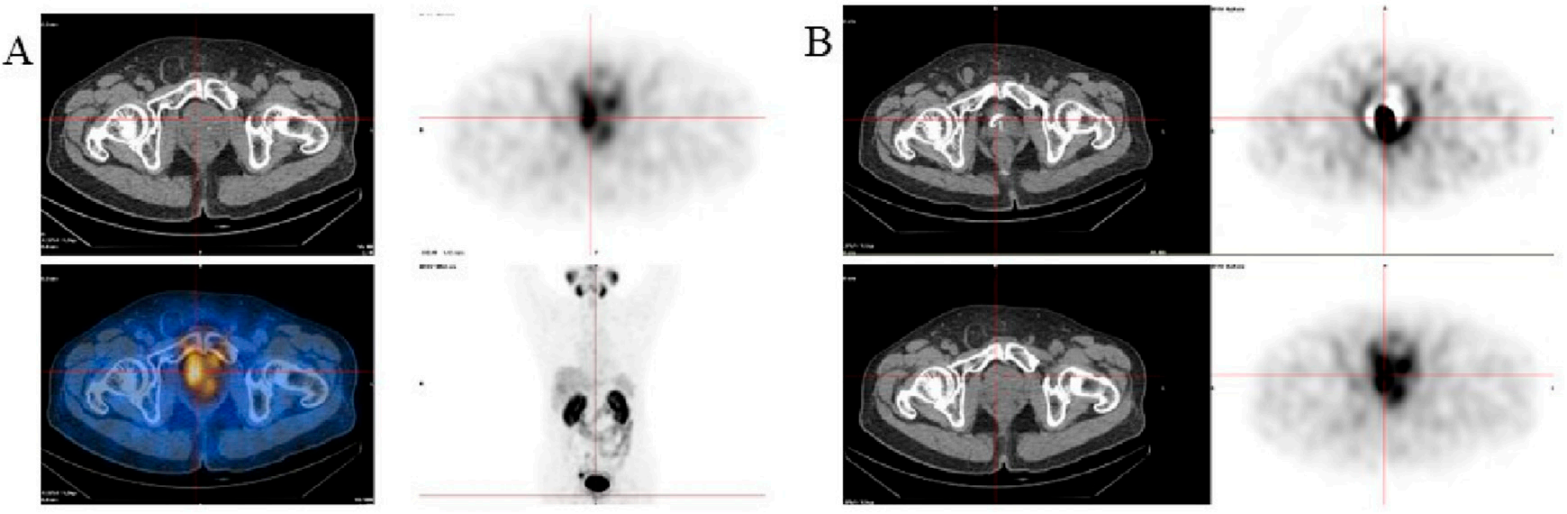
Representative PSMA images of a patient receiving darolutamide-based triplet therapy followed by high-dose intensity-modulated radiotherapy (IMRT). **(A)** PSMA IMAGING after completion of triplet therapy showing residual focal uptake within the prostate, indicating partial metabolic response after systemic treatment. **(B)** Follow-up PSMA IMAGING 3 months after high-dose IMRT demonstrating a marked reduction in SUVmax and metabolic activity of the primary lesion, consistent with continued PSA decline and durable local control.

Based on MDT assessment, eight patients (47.1%) underwent focal surgery for prostate including prostatectomy (some with pelvic lymph node dissection) or TURP, while nine patients (52.9%) received high-dose intensity-modulated radiotherapy (IMRT) (Figure 2), of whom two also received stereotactic body radiotherapy (SBRT) to bone metastases. All focal treatments were completed successfully, with no perioperative deaths or severe radiotherapy-related complications.

### PSA response and survival outcomes

The median baseline PSA was 39.7 ng/mL (IQR, 23.0–163.0). Following systemic therapy, 94.1% (16/17) of patients achieved a PSA90 response, and 82.4% (14/17) achieved a PSA ≤0.2 ng/mL (Table 2). At the final follow-up in August 2025, the median follow-up duration was 16 months (range, 11–20 months).

**TABLE 2:** Treatment response and outcomes.

Variable	Result (range)	95% CI
Median chemotherapy cycles	5 (range 4–6)	—
Median duration of darolutamide treatment (months)	14 (range 9–18)	—
Ongoing darolutamide at last follow-up	15 (88.2%)	—
Combined with ^177Lu therapy	1 (5.9%)	—
Combined with olaparib	1 (5.9%)	—
Changed to other therapies due to progression	2 (11.8%)	—
Local treatment modalities	Focal surgery: 8 (47.1%); high-dose IMRT: 9 (52.9%) (including 2 cases with SBRT to bone metastases)	—
PSA50 response rate	17 (100.0%) (PSA decline range: 73.1%–100.0%)	—
PSA90 response rate	16 (94.1%) (PSA decline range: 73.1%–100.0%)	—
PSA ≤0.2 ng/mL	14 (82.4%)	—
12-month/18-month PSA-PFS rate^a^	88%/65%	72%–100%/45%–85%
12-month/18-month rPFS rate^a^	94%/76%	83%–100%/58%–94%
Median PSA-PFS (months)	Not reached (range 8.0–16.0)	—
Median rPFS (months)	Not reached (range 11.0–16.0)	—
Median follow-up (months)	16.0 (range 11.0–20.0)	—

^a^ Kaplan-Meier analysis.

Abbreviations: IMRT, intensity-modulated radiotherapy; SBRT, stereotactic body radiotherapy; PSA-PFS, prostate-specific antigen progression-free survival; rPFS, radiographic progression-free survival; ARI, androgen receptor inhibitor.

**FIGURE 3:**
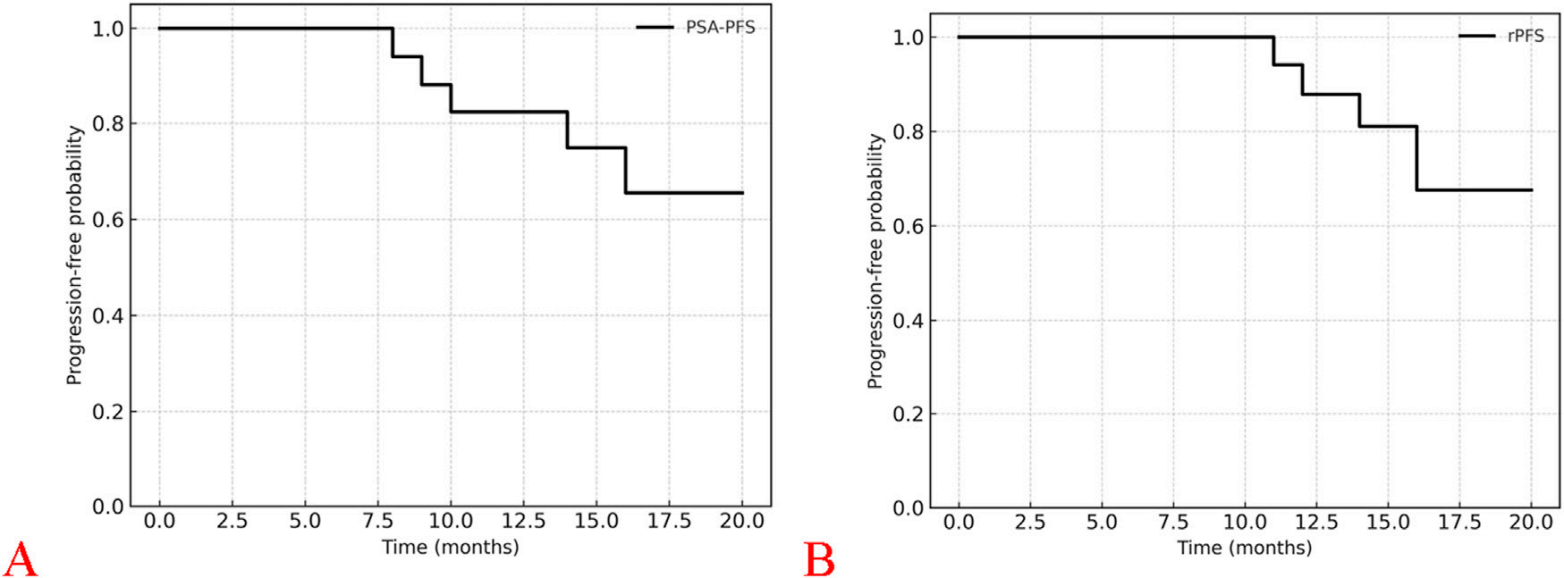
Kaplan–Meier curves of progression-free survival outcomes in patients receiving darolutamide-based triplet therapy combined with PSMA IMAGING-guided focal treatment. **(A)** Kaplan–Meier curve of PSA progression-free survival (PSA-PFS). **(B)** Kaplan–Meier curve of radiographic progression-free survival (rPFS).

Kaplan–Meier survival analysis was performed to estimate PSA progression-free survival (PSA-PFS) and radiographic progression-free survival (rPFS). The Kaplan–Meier curves are presented in Figure 3. The 12- and 18-month PSA-PFS rates were 88% (95% CI, 72%–100%) and 65% (95% CI, 45%–85%), respectively; the corresponding rPFS rates were 94% (95% CI, 83%–100%) and 76% (95% CI, 58%–94%). Median PSA-PFS and rPFS were not reached at the data cutoff, indicating that the majority of patients remained progression-free throughout the 18-month follow-up period.

**FIGURE 4:**
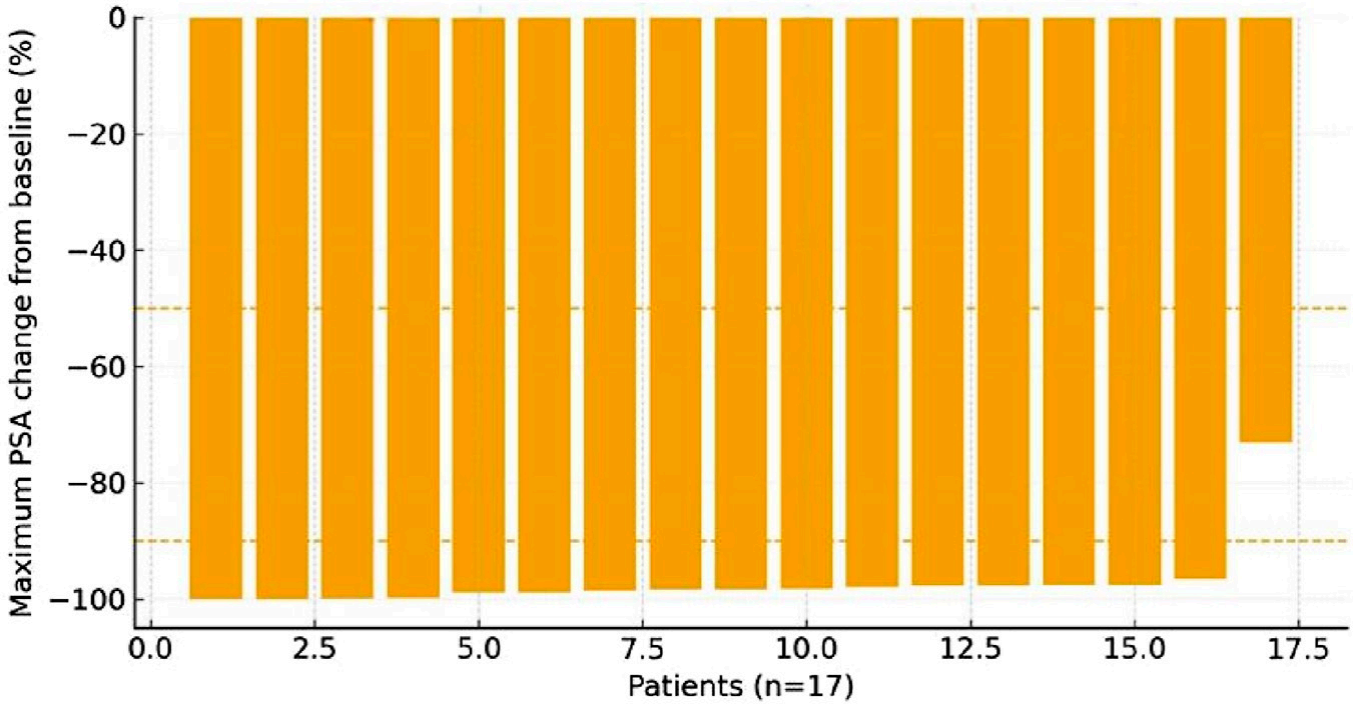
Waterfall plot illustrating individual PSA responses to darolutamide-based triplet therapy combined with PSMA IMAGING-guided focal treatment. Abbreviations: PSA, prostate-specific antigen; PSMA, prostate-specific membrane antigen; ADT, androgen-deprivation therapy.

Among the patients who experienced progression, the median PSA-PFS was 10 months (range, 8–16 months), and the median rPFS was 13 months (range, 11–16 months). A waterfall plot (Figure 4) illustrates the maximum percentage change in PSA from baseline for each patient, highlighting inter-patient variability in treatment response. Most patients demonstrated a durable and profound PSA decline following darolutamide-based triplet therapy combined with PSMA-guided focal intervention.

### Adverse events

Treatment-related adverse events were predominantly grade 1–2 myelosuppression (n = 11, 64.7%) and fatigue (n = 8, 47.1%) (Table 3). Two patients (11.8%) developed grade 3 neutropenia requiring temporary treatment interruption, both of whom recovered with supportive care; no grade 4 or higher toxicities were observed. The median time to neutropenia nadir was approximately 10–12 days after each docetaxel cycle, and hematologic recovery typically occurred within 7 days with granulocyte colony-stimulating factor (G-CSF) support. No cumulative hematologic toxicity or treatment discontinuation due to cytopenia was reported during sequential cycles of darolutamide plus docetaxel. Mild to moderate gastrointestinal symptoms (nausea, constipation) were common and manageable with symptomatic treatment, with no grade ≥3 gastrointestinal toxicity observed. Focal therapy was well tolerated, with no severe acute or late complications, and all patients completed treatment as planned.

**TABLE 3:** Adverse events.

Adverse event	n (%)	Grade	Management
Leukopenia	11 (64.7%)	G2	Symptomatic supportive care, continued chemotherapy
Myelosuppression	2 (11.8%)	G3	Paused chemotherapy, G-CSF support
Gastrointestinal reactions (nausea/constipation)	Multiple cases (see raw data)	G1–G2	Symptomatic treatment
Fatigue	8 (47.1%)	G1–G2	Rest and supportive care
Mild urinary frequency related to local therapy	1 (5.9%)	G1	Observation, no specific intervention
Mild rectal irritation symptoms related to local therapy	1 (5.9%)	G1	Symptomatic relief
Other severe adverse events (≥G4)	0	—	—

Abbreviations: G-CSF, granulocyte colony-stimulating factor; G, grade; IMRT, intensity-modulated radiotherapy; SBRT, stereotactic body radiotherapy.

## Discussion

This multicenter, real-world study from China evaluated the efficacy and safety of triplet therapy with darolutamide, docetaxel, and androgen deprivation therapy (ADT), combined with PSMA IMAGING–guided focal treatment, in patients with metastatic hormone-sensitive prostate cancer (mHSPC). Our results demonstrated that this approach achieved rapid and profound PSA responses (PSA90 rate >90%) in most patients, with favorable tolerability. These findings are consistent with the ARASENS trial results, which established triplet therapy as a standard of care in mHSPC (Kyriakopoulos et al., 2018), and suggest that its clinical benefits are reproducible in Chinese practice.

Compared with systemic therapy alone, our study emphasizes an integrated strategy of “systemic control plus precise local eradication.” Triplet therapy rapidly reduced systemic tumor burden, allowing most patients to achieve deep PSA responses after chemotherapy. Subsequent PSMA IMAGING imaging allowed precise detection of residual active disease, enabling MDT to selectively perform prostatectomy or high-dose radiotherapy on the primary tumor and/or oligometastatic lesions. The sequence and timing of these interventions were deliberately structured to optimize therapeutic synergy. The timing of focal therapy, scheduled 4–6 weeks after completion of docetaxel, was intentionally chosen based on both biological and clinical considerations. This interval allowed sufficient hematologic recovery and resolution of chemotherapy-related inflammation, thereby minimizing perioperative risks and ensuring patient fitness for local intervention. Performing PSMA IMAGING after a 4–6 weeks interval also reduced the likelihood of treatment-related inflammatory uptake, improving the accuracy of residual disease assessment and target delineation. Moreover, previous multimodal studies, including the STAMPEDE and HORRAD trials, have suggested that initiating local therapy after achieving initial systemic control enhances therapeutic synergy and optimizes long-term disease suppression. Collectively, this sequence ensured safer implementation and more precise patient selection for focal treatment. This phased and individualized intervention model may help prevent local progression, delay systemic relapse, and offer a subset of patients the opportunity for durable remission, as suggested by prior studies on local treatment in mHSPC (Boeve et al., 2019; Parker et al., 2018).

The biological rationale for combining systemic and focal treatments lies in the complementary mechanisms of disease control. Systemic therapy with androgen deprivation, chemotherapy, and androgen receptor inhibition targets disseminated microscopic disease and circulating tumor cells, while focal ablation of dominant lesions may eradicate potential sources of resistant clones and reduce tumor-derived immunosuppressive signaling. Local control of the primary or oligometastatic lesions has been associated with improved immune surveillance and delayed systemic progression in both preclinical and clinical studies (Parker et al., 2018; Fendler et al., 2016; Ost et al., 2018). Therefore, integrating PSMA-guided focal therapy into systemic treatment may create a synergistic effect, enhancing overall tumor control and postponing the emergence of castration resistance.

In recent years, several clinical studies (such as the STAMPEDE and HORRAD trials) have demonstrated that, in patients with low metastatic burden mHSPC, combining systemic therapy with radiotherapy to the primary tumor can improve overall survival or delay disease progression (Boeve et al., 2019; Parker et al., 2018). For patients with oligometastatic disease, metastasis-directed therapy using SBRT has also shown positive effects in delaying the need for systemic therapy and improving quality of life (Ost et al., 2015). Therefore, integrating local interventions on the basis of triplet systemic therapy, particularly when guided by precision imaging–based patient selection, may further enhance the depth and durability of disease control.

The incorporation of PSMA IMAGING was a key feature of this study. Compared with conventional imaging, PSMA IMAGING offers superior sensitivity for detecting low-volume or occult disease, thereby improving the precision of focal treatment planning (Hofman et al., 2020; Bashir et al., 2019). In this multicenter study, both ^68Ga-PSMA PET/CT and ^99mTc-PSMA SPECT/CT were utilized across different centers. Although ^68Ga-PSMA PET/CT generally provides higher spatial resolution and sensitivity than ^99mTc-PSMA SPECT/CT, all imaging evaluations were interpreted by experienced nuclear medicine physicians, and final treatment decisions were made through multidisciplinary team (MDT) discussions. These measures helped to minimize the potential variability in lesion detectability between modalities and reduce its impact on treatment selection. Previous evidence indicates that treating the primary tumor in mHSPC can improve survival (Boeve et al., 2019; Parker et al., 2018), and image-guided focal interventions based on high-sensitivity molecular imaging may further enhance disease control depth. Although our study supports this hypothesis to some extent, the limited sample size and follow-up duration preclude definitive conclusions regarding long-term survival benefits.

The safety profile observed in our cohort was favorable. Darolutamide’s low blood–brain barrier penetration may reduce the incidence of fatigue and cognitive impairment compared with other androgen receptor inhibitors (Kyriakopoulos et al., 2018; Fizazi et al., 2019). Most hematologic toxicities and gastrointestinal side effects were mild-to-moderate and manageable, and focal therapies did not cause severe perioperative or radiotherapy-related complications. This is clinically relevant in mHSPC, where long-term systemic treatment is often required, as minimizing treatment-related toxicity can help maintain adherence and quality of life.

In the ARASENS trial, the most common adverse events included neutropenia (39.3% for grade ≥3), alopecia (40.5%), and fatigue (33.1%), while serious adverse events occurred in 44.8% of patients and treatment-related deaths in 1.5%. In contrast, our real-world cohort showed a lower incidence of hematologic and non-hematologic toxicities, with grade 3 myelosuppression in only 11.8% of patients and no grade ≥4 events. Fatigue was reported in 47.1% of cases but was mild-to-moderate and manageable. No treatment-related deaths or serious complications from focal therapy were observed. This lower toxicity profile may be attributed to the small sample size, selection of relatively fit patients, and close multidisciplinary monitoring, indicating that darolutamide-based triplet therapy combined with focal treatment is well tolerated in Chinese clinical practice. Moreover, hematologic toxicities were transient and well managed. No cumulative toxicity was observed throughout docetaxel administration in combination with continuous darolutamide, suggesting that this regimen does not exacerbate chemotherapy-related myelosuppression. Gastrointestinal adverse events were mild and reversible, indicating a favorable overall tolerability profile.

Despite encouraging results, this study has several limitations. The relatively small sample size and retrospective design introduce potential selection bias, and the follow-up duration was insufficient to observe median rPFS or OS in most patients. Another limitation is the lack of formal control cohorts treated with ADT alone or ADT plus docetaxel, which limits the ability to directly compare the efficacy of the triplet regimen and isolate the independent contribution of PSMA-guided focal therapy. Furthermore, the use of two different imaging modalities (^68Ga-PSMA PET/CT and ^99mTc-PSMA SPECT/CT) across participating centers may have introduced minor variability in lesion detection and patient selection. In addition, due to incomplete post-treatment PSMA imaging data for some patients, a quantitative SUV-based response analysis could not be performed. This prevented direct correlation of biochemical and imaging endpoints. Future prospective studies incorporating standardized serial PSMA IMAGING assessments (including SUVmax and lesion-based response) will be essential to validate and complement PSA-based endpoints for a more comprehensive evaluation of therapeutic efficacy.

Although the majority of our patients had low-to intermediate-volume metastatic disease, this pattern reflects the real-world subgroup most frequently selected for focal therapy after systemic triplet induction. Importantly, all patients underwent PSMA IMAGING before focal intervention, not as an inclusion filter, thereby minimizing selection bias. These limitations primarily reflect the early clinical adoption phase of darolutamide-based triplet therapy in China following its national insurance inclusion in late 2023. Nevertheless, our findings provide valuable real-world evidence supporting the feasibility and safety of combining systemic triplet therapy with PSMA-guided focal treatment. To address the lack of control groups and small sample size, an expanded multicenter registry has been initiated at these centers. This registry includes matched cohorts receiving ADT alone and ADT plus docetaxel, enabling propensity score–matched analyses of PSA kinetics, rPFS, and overall survival. These forthcoming data will help validate and refine the clinical benefit of this combined “systemic plus local” multimodal strategy. We acknowledge that multivariable adjustment or propensity score analyses were not performed in this exploratory study because of the limited sample size. Nevertheless, standardized inclusion criteria, endpoint definitions, and STROBE-compliant data collection helped reduce potential selection and measurement bias. Future prospective studies with larger cohorts will incorporate multivariable and sensitivity analyses to confirm the robustness of these findings.

## Conclusion

This multicenter real-world study suggests that PSMA-guided focal therapy combined with darolutamide-based triplet therapy achieves a high rate of deep PSA responses in selected patients with metastatic prostate cancer while maintaining favorable safety and tolerability. This integrated approach appears feasible and potentially beneficial in clinical practice; however, its impact on long-term survival outcomes warrants further validation in large-scale, prospective, randomized controlled trials.

## Data Availability

The original contributions presented in the study are included in the article/supplementary material, further inquiries can be directed to the corresponding authors.
